# Wound healing and pain evaluation following diode laser surgery vs. conventional scalpel surgery in the surgical treatment of oral leukoplakia: a randomized controlled trial

**DOI:** 10.3389/froh.2025.1568425

**Published:** 2025-03-03

**Authors:** Mariana de Pauli Paglioni, Caique Mariano Pedroso, Isabel Schausltz Pereira Faustino, Pablo Agustin Vargas, Mario Fernando de Goes, Manoela Domingues Martins, Márcio Ajudarte Lopes, Alan Roger Santos-Silva

**Affiliations:** ^1^ Piracicaba Dental School, University of Campinas (UNICAMP), Piracicaba, Brazil; ^2^ Department of Oral Pathology, School of Dentistry, Universidade Federal do Rio Grande do Sul—UFRS, Porto Alegre, Brazil

**Keywords:** oral leukoplakia, diode laser, surgical excision, pain, healing, randomized clinical trial

## Abstract

**Objectives:**

This study aimed to compare patient-reported pain scores and clinician-assessed healing outcomes following the treatment of oral leukoplakia (OL) with a diode laser vs. a conventional scalpel.

**Methods:**

A randomized, double-blind clinical trial (Brazilian Clinical Trials Registry (RBR-7pgcyq) was conducted involving histopathologically confirmed OL patients. Participants were randomly allocated to undergo treatment with either a diode laser or a scalpel. Pain was assessed at 24 h, 48 h, and 7 days using the Visual Analog Scale (VAS), while healing outcomes were clinically evaluated at 7 days, 1-month, and 3-months post-treatment using the Vancouver Scar Scale. Statistical analyses included the Mann–Whitney *U*-test for comparing pain and healing scores between interventions. Friedman test also was used to analyze healing progress over time.

**Results:**

64 patients were analyzed (33 in diode laser and 31 in scalpel group). No significant differences in pain scores were observed between the treatment groups at 24 h (*p* = 0.75), 48 h (*p* = 0.92), or 7 days (*p* = 0.44). Overall, pain levels varied significantly by OL location at 24 h (*p* = 0.001), 48 h (*p* = 0.01), and 7 days (*p* = 0.03), with tongue lesions associated with significantly higher pain compared to gingival lesions at 24 h (*p* = 0.005) and 48 h (*p* = 0.01), as well as compared to palatal lesions at 24 h (*p* = 0.01). Laser group showed significantly better healing compared to the scalpel group at 7 days (*p* = 0.01), with no significant differences observed at 1 month (*p* = 0.67) or 3 months (*p* = 0.25). Healing outcomes improved significantly over time in both arms (*p* < 0.001).

**Conclusions:**

There was no significant difference between the diode laser and scalpel treatment arms regarding post operative pain scores. Diode lasers represent better healing at the first week post treatment, but with no differences over time. These findings support the use of either modality as viable management options for OL.

**Clinical Trial Registration:**

Brazilian Clinical Trials Registry, identifier (RBR-7pgcyq).

## Introduction

Oral leukoplakia (OL) is the most common oral potentially malignant disorder, characterized by the presence of a white plaque that cannot be attributed to any other condition (1). A definitive OL diagnosis requires histopathological examination, and surgical intervention is typically recommended when oral epithelial dysplasia is detected (2). The primary goal of OL surgical intervention is to reduce the risk of malignant transformation (3). Surgical excision, using either a scalpel or laser therapy, remains one of the most widely adopted approaches (3, 4).

Conventional scalpel-based surgical treatment poses several challenges, including intraoperative bleeding and the potential for significant postoperative complaints, particularly in larger lesions (4). Despite these challenges, previous evidence indicates that the risk of malignant transformation is comparable between operated and non-operated patients, raising questions about the overall efficacy of traditional surgical methods (5).

To address these challenges, laser surgery has emerged as a promising alternative for the treatment of OL (6–9). The literature suggests that lasers offer several advantages over scalpel, including superior hemostasis, reduced postoperative discomfort, and lower risk of infection (10). Furthermore, laser surgery promotes healing by secondary intention, making it particularly suitable for large or anatomically challenging lesions (7, 8). These advantages underscore the potential of laser to address the complexities associated with traditional surgical approaches.

In this context, exploring alternatives techniques, such as diode laser, is crucial for advancing OL management. In addition, the evidence concerning which surgical method is more efficient to control pain and healing remains unclear. So far, no RCTs have been undertaken to investigate the benefits of different treatment modalities. Therefore, this study aimed to compare patient-reported outcomes related to pain and observer recorded healing outcomes between diode laser therapy and scalpel excisions in the treatment of OL. We tested the following null hypothesis: there is no significant difference in pain and healing between treatment groups of oral leukoplakia managed with a diode laser and scalpel excision.

## Materials and methods

### Trial design

This is part of an ongoing prospective randomized, double-blind clinical trial with a 1:1 allocation ratio (11). The experimental design adheres to guidelines outlined in the Consolidated Standards of Reporting Trials (CONSORT) (12). The study was approved by the Research Ethics Committee of the Piracicaba Dental School, University of Campinas, Brazil, (CAAE:20196919.3.0000.5418) and registered with the Brazilian Clinical Trials Registry (RBR-7pgcyq- Trial registry number—registered and approved on 03 November, 2020).

### Eligibility criteria for participants

The inclusion criteria consisted of adult new patients (>18 years) presenting with white plaques on the oral mucosa, with no restriction on lesion size. The diagnosis of OL followed the guidelines and definitions established by the WHO Collaborating Centre for Oral Cancer (1). Lesions located on the tongue, mouth floor, retromolar region, buccal mucosa, soft palate, or gingiva were included. Homogeneous and non-homogeneous OL were included. Incision biopsies were performed to confirm the diagnosis of OL and patients with histopathologically confirmed white plaques attributed to other conditions were excluded. Patients with proliferative verrucous leukoplakia were excluded. Additionally, individuals with a history of previous oral carcinomas, chemotherapy, or radiotherapy in the head and neck region were not eligible for inclusion.

### Study intervention

Following OL diagnosis, the assigned treatment was implemented. All surgical procedures were performed by a single surgeon. Local anesthesia was administered using 2% lidocaine combined with 1:100,000 adrenaline (Alphacaine 100, DFL, Rio de Janeiro, Brazil). In both treatment groups, the lesions were excised with a 3 mm safety margin, ensuring that the underlying muscle tissue was included in the depth of the excision. A previous study determined that a 3 mm margin is optimal for minimizing the risk of lesion recurrence (13). In the treatment group, diode laser therapy was performed using a high-power diode laser (DMC U.S.A, Plantation, FL, USA; 940 nm, 4 watts, pulsed mode, 10 ms repetition, frequency 100 Hz). Appropriate safety protocols, including the use of lasers protective glasses and restricted access to the surgical area, were followed. In the control group, conventional scalpel excision was carried out. The lesions were excised using a disposable carbon steel scalpel blade (No. 15). After excision, the surgical sites were closed with sterile, absorbable sutures (Polyglactin 910, composed of glycolide 90% and lactide 10%, Shalon Medical, Goiânia, Brazil). Gingival sutures were not applied when infeasible in control group; instead, compression was used.

### Outcomes

The primary outcome assessed was postoperative healing, while the secondary outcome was pain. Pain was assessed at 24 h, 48 h, and 7 days post-procedure. Patients rated their pain using a visual analog scale (VAS) ranging from 0 (no pain) to 10 (the worst pain experienced). Healing was evaluated at 1 week, 1 month, and 3 months post-procedure using Vancouver Scar Scale (VSS) (14). Three criteria (contour, distortion, and texture) were assessed, with scores ranging from 1 to 4. Higher scores indicated poorer healing outcomes.

### Sample size

The sample calculation was carried out considering a 20% difference in postoperative healing between the groups (laser and scalpel) found in previous study (15). According to a standard deviation of approximately 40% of the mean and with a power of 80% and a significance level of 5% (BioEstat version 5.3), the number of 30 patients per group was estimated.

### Recruitment, blinding, randomization, allocation concealment, and implementation

Patient recruitment was conducted through clinical examination of individuals referred to the Oral Medicine Service at the Piracicaba Dental School, University of Campinas, São Paulo, Brazil, for evaluation of white plaques in the oral mucosa. Enrollment of patients started in 2021. Following clinical examination and the OL diagnosis (confirmed via incisional biopsy and histological analysis), eligible patients were invited to participate in the study. Written informed consent was obtained from all patients prior to inclusion. A double-blind methodology was employed, with blinding implemented at two levels: patients and outcome evaluators. Randomization was conducted by a researcher not directly involved in the study, using a randomization list generated in Microsoft Excel. The treatment assignments were recorded on paper, sealed in opaque envelopes, and securely stored. The surgeon determined the treatment by opening an envelope immediately prior to the procedure.

### Statistical analysis

The Mann–Whitney *U*-test was used to compare pain levels (24 h, 48 h, and 7 days post-surgery) and healing (7 days, 1 month, and 3 month) between the scalpel and laser surgery treatment. Spearman's correlation coefficient was applied to assess the correlation between pain and patient age. The Kruskal–Wallis test was used to determine differences in pain based on lesion location, followed by Dunn's *post-hoc* test with Bonferroni correction. For wound healing data from the laser and scalpel treatments, the Friedman test was used to evaluate changes in healing parameters (contour, distortion, and texture) over time, considering repeated measures in both treatment groups. The Wilcoxon test with Bonferroni adjustment was applied to perform pairwise comparisons of healing between different time points, controlling for type I error. The Kruskal–Wallis test was also employed to investigate the influence of lesion location on healing. A significance level of 5% was set for all analyses, which were performed using RStudio statistical software.

## Results

### Participant flow

**Figure 1:**
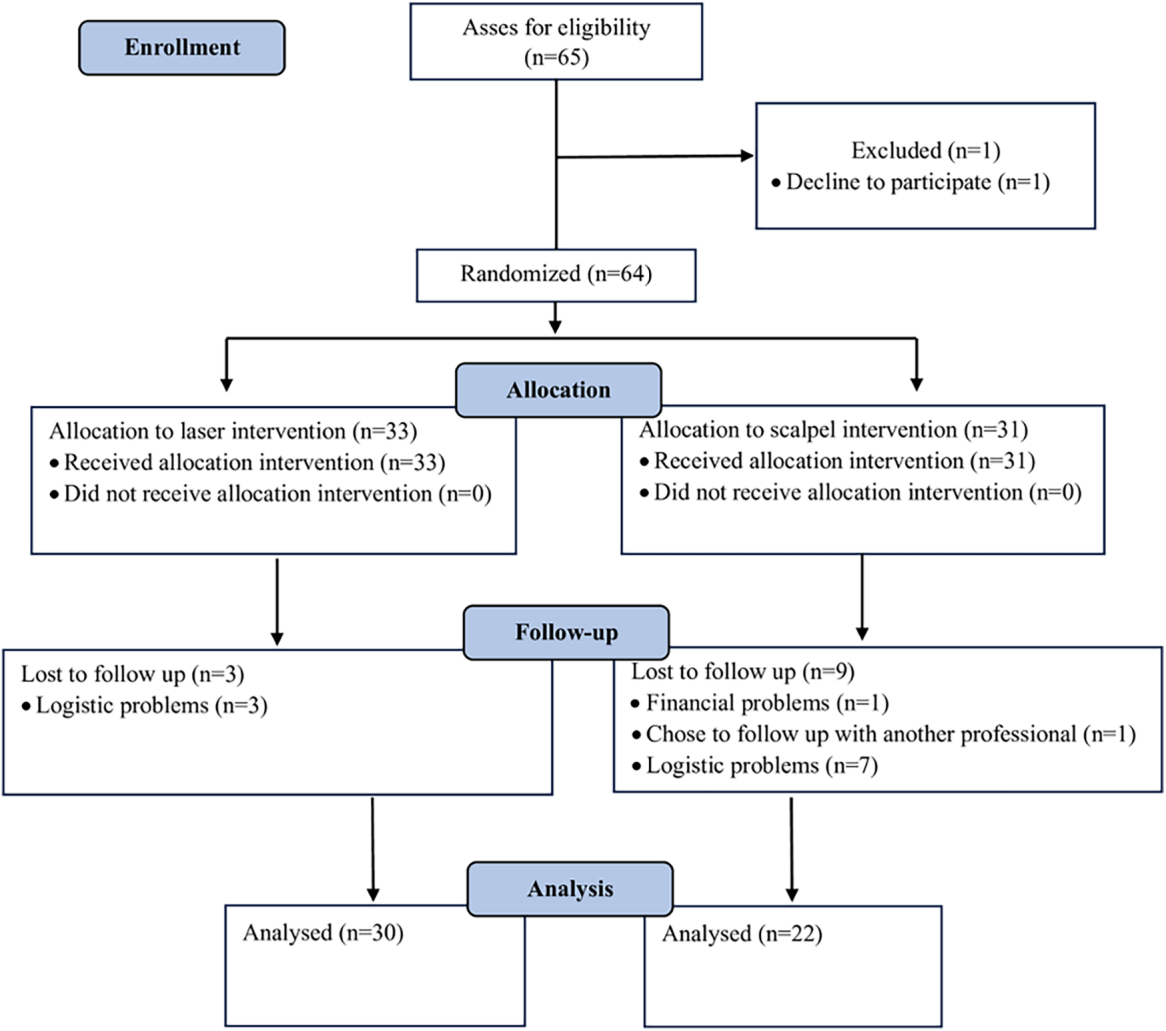
Flow diagram of the recruitment, randomization, and allocation concealment process.

The flow diagram illustrating the recruitment, randomization, and allocation concealment process is shown in Figure 1. A total of 64 patients were included in the study, with 33 allocated to the laser surgery arm and 31 to the scalpel arm. All participants completed the 7-day postoperative pain evaluation and the 1-month healing assessment. 12 participants dropped out of the 3-month healing evaluation, (3 in the laser arm and 9 in the scalpel arm). These participants were unable to complete the 3-month evaluation primarily due to difficulties in attending review appointments (cited as financial or logistical issues).

### Baseline data

**Figure 2:**
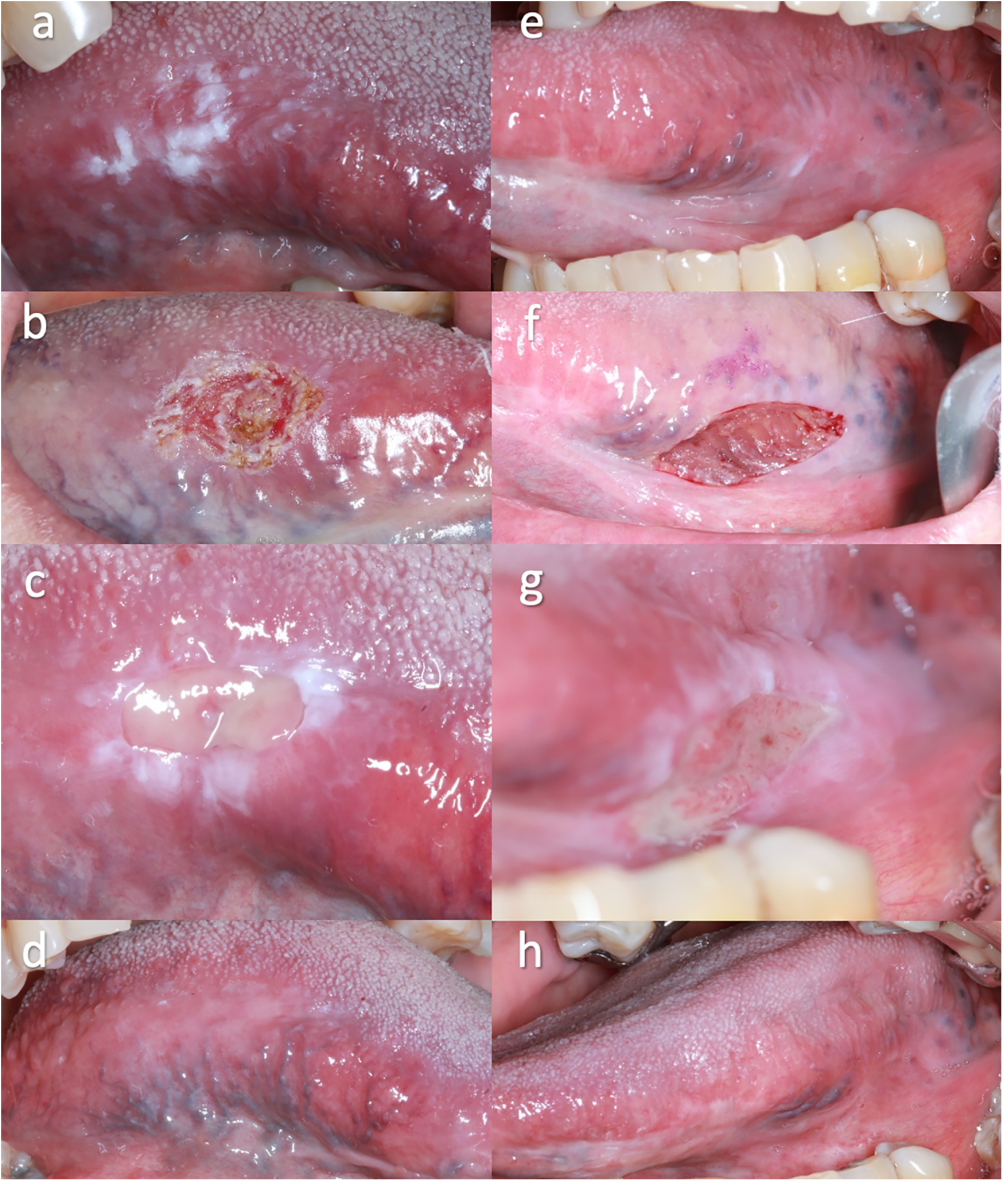
Surgical procedures and healing outcomes for the laser group **(a–d)** and scalpel group **(e–h)**. **(a,e)** Initial clinical appearance of oral leukoplakia. Excision was performed using laser **(b)** and scalpel **(f)**, showing the immediate postoperative appearance. **(c,g)** 7 days postoperatively, a fibrinopurulent membrane was visible at the site where the oral leukoplakia was removed. **(d,h)** 3-months postoperatively, complete healing of the lesions was observed in both treatment groups.

The laser surgery group comprised 33 patients, including 13 women and 20 men, with a mean age of 60.7 years (range: 33–73 years). The proportion of lesions in laser group were located on tongue (39.3%), buccal mucosa (21.2%), palate (21.2%), gingiva (12.1%), and retromolar (6.0%). The scalpel group comprised 31 patients, including 12 women (38.7%) and 19 men (61.3%), with a mean age of 59.5 years (range: 23–81 years). The distribution of lesions in scalpel group were: tongue (61.2%), palate (19.3%), buccal mucosa (16.1%), and gingiva (3.2%). Surgical procedures and healing outcomes for the laser and scalpel groups are illustrated in Figure 2.

### Pain outcome

**Figure 3:**
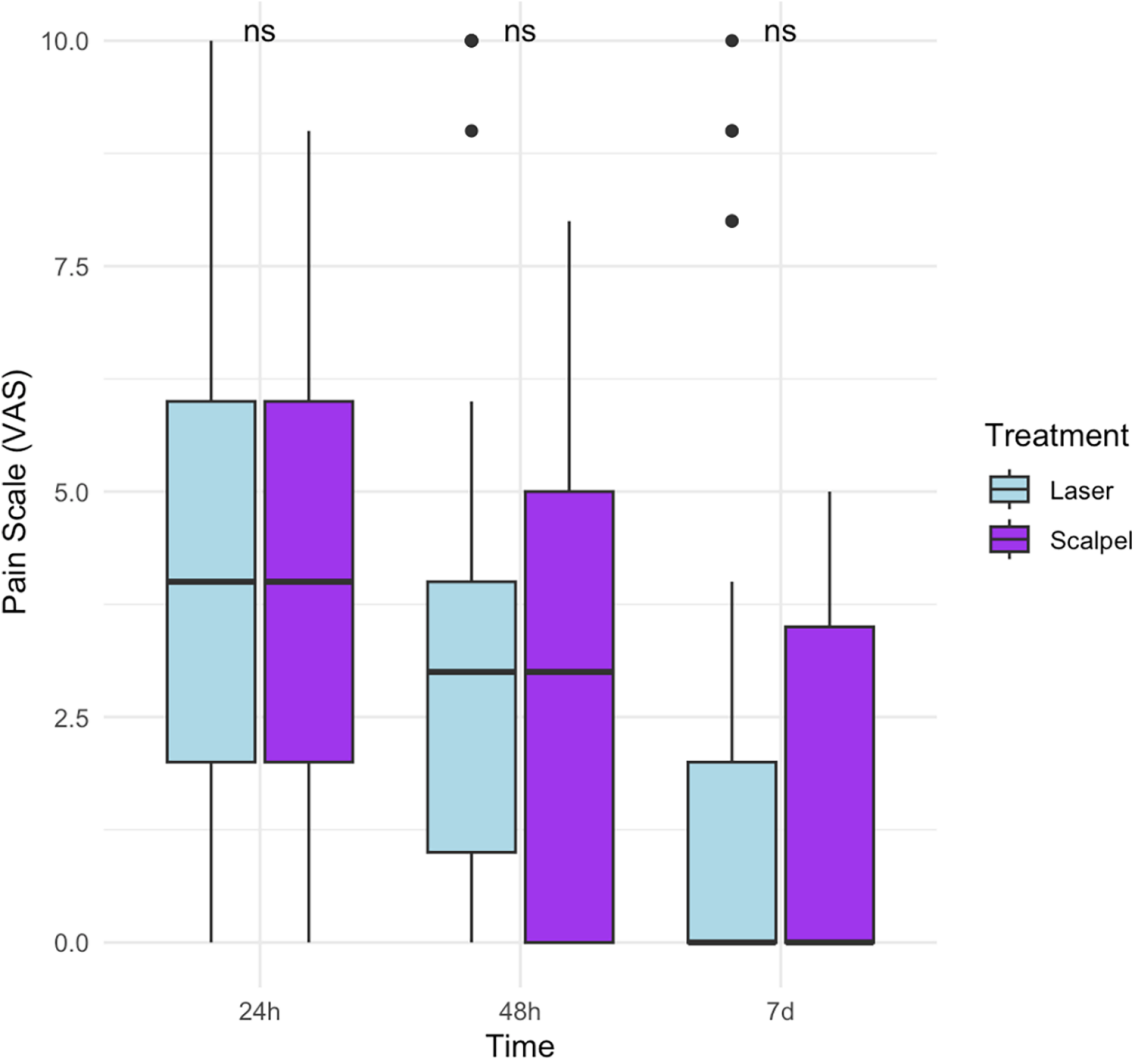
Analysis of postoperative pain in laser and scalpel.

The mean pain scores reported by patients (based on the VAS Scale) in the laser arm was 4.06 (±3.16) at 24 h after the procedure, 3.42 (±3.21) at 48 h, and 1.72 (±3.14) at 7 days (Table 1). In the scalpel arm, the mean pain scores reported was 4.12 (±2.76) at 24 h, 3.19 (±2.59) at 48 h, and 1.58 (±1.79) at 7 days (Table 1). The analysis of postoperative pain between the laser and scalpel treatment groups for OL revealed no statistically significant differences at 24 h (*p* = 0.75), 48 h (*p* = 0.92), or 7 days (*p* = 0.44) (Figure 3).

**Table 1:** Pain intensity in visual analog scale—mean (standard deviation).

VAS	Scalpel	Laser	*p-value
24 h	4.12 (±2.76)	4.06 (±3.16)	0.75
48 h	3.19 (±2.59)	3.42 (±3.21)	0.92
7 days	1.58 (±1.79)	1.72 (±3.14)	0.44

* Mann–Whitney *U*-test.

**Figure 4:**
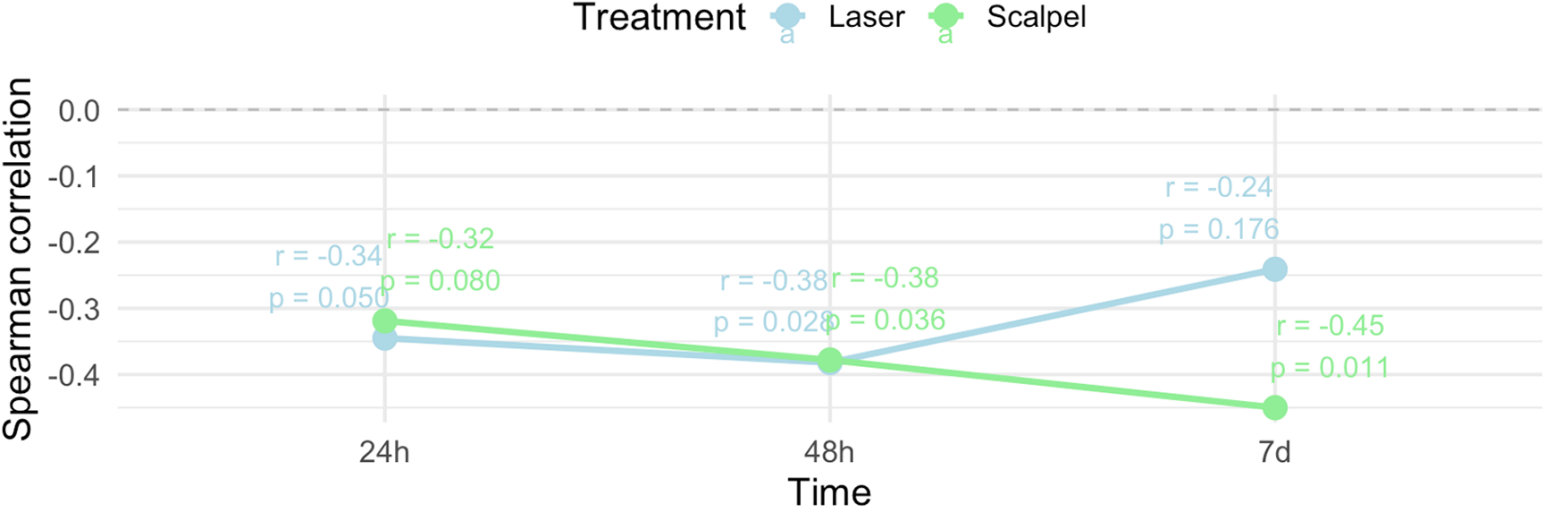
Correlation between pain and age in both treatment groups combined.

A significant negative correlation for both groups combined was observed between pain and age at 24 h (rho = −0.322, *p* = 0.009) and 48 h (rho = −0.373, *p* = 0.002), indicating that older patients experienced lower pain levels (Figure 4). Additionally, pain levels for both groups combined varied significantly by lesion location at 24 h (*p* = 0.003) and 48 h (*p* = 0.030), though no significant difference was observed at 7 days (*p* = 0.057). Dunn's *post-hoc* tests with Bonferroni correction revealed that at 24 h, pain was significantly higher for lesions on the tongue compared to those on the gingiva and palate, and at 48 h, pain was higher for lesions on the tongue compared to those on the gingiva.

### Healing outcome

**Figure 5:**
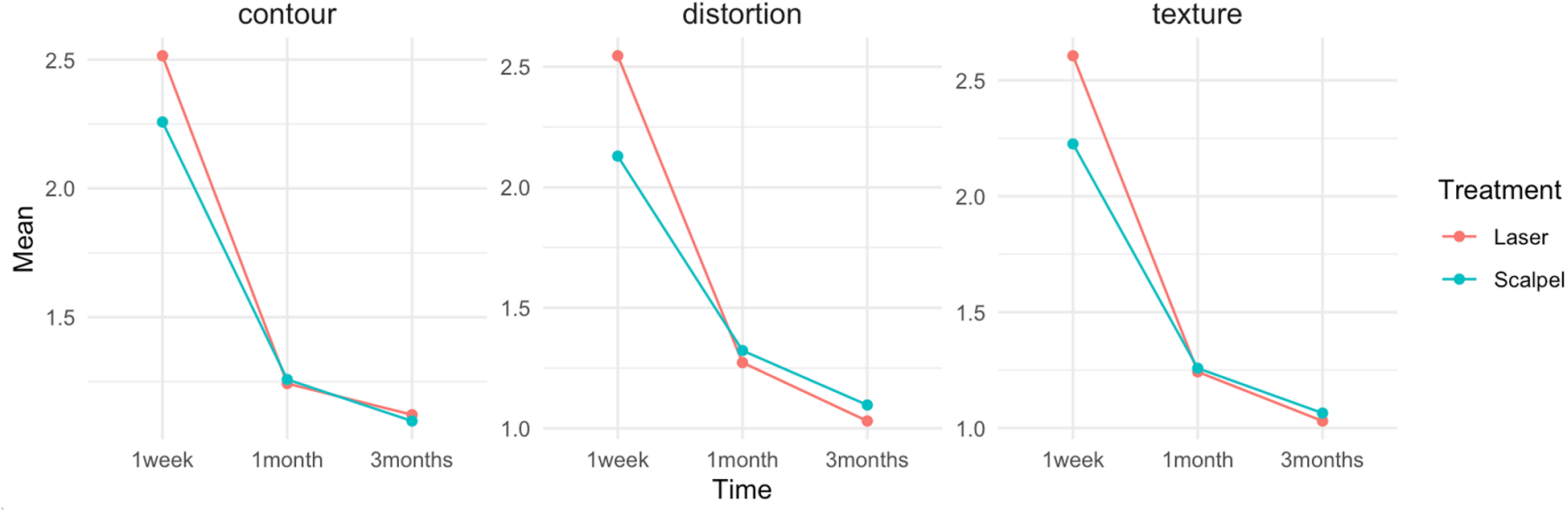
Healing parameters (contour, distortion, and texture) over time for both laser and scalpel treatments.

The laser treatment arm demonstrated a mean contour score of 2.51, a mean distortion score of 2.54, and a mean texture score of 2.60 at 7 days post-treatment. By 1-month post-treatment, these scores improved to 1.33 for contour, 1.30 for distortion, and 1.27 for texture. At 3 months, the scores further decreased, with contour averaging 1.13, distortion 1.03, and texture 1.03 (Table 2). In the scalpel treatment arm, the mean contour score at 7 days post-treatment was 2.25, with a mean distortion score of 2.12 and a mean texture score of 2.22. At 1-month post-treatment, the mean contour score improved to 1.25, distortion to 1.32, and texture to 1.25. By 3 months, the scores further declined to 1.14 for contour, 1.04 for distortion, and 1.04 for texture (Table 2). The Mann–Whitney tests revealed a significant difference between the diode laser and scalpel groups at 7 days (*p* = 0.01), but no significant differences at 1 month (*p* = 0.67) and 3 months (*p* = 0.25). Significant differences were observed in healing parameters (contour, distortion, and texture) over time for both laser and scalpel treatments (*p* < 0.001 for both). Improvements were most pronounced between the first week and the third month in both treatment groups, but there were no significant differences between the first and third months for most of the parameters (*p* = 0.09) (Figure 5). The anatomical location of the lesion (buccal mucosa, palate, and tongue) had no significant effect on healing outcomes for any parameter, with all *p*-values exceeding 0.05.

**Table 2:** Vancouver scar Scale (14) at the 7 days, 1- and 3-months follow-up.

	Laser group			Scalpel group			*p-value
	Contour	Distortion	Texture	Contour	Distortion	Texture	
7 days							
Mean	2.5 (±0.79)	2.5 (±0.79)	2.6 (±0.7)	2.2 (±0.4)	2.1 (±0.3)	2.2 (±0.4)	0.01
1 month							
Mean	1.2 (±0.6)	1.2 (±0.6)	1.2 (±0.6)	1.2 (±0.5)	1.3 (±0.4)	1.2 (±0.4)	0.67
3 months							
Mean	1.1 (±0.3)	1.0 (±0.1)	1.0 (±0.1)	1. 0(±0.3)	1.0 (±0.3)	1.0 (±0.2)	0.25

* Mann–Whitney *U*-test.

## Discussion

Our findings did not yield sufficient evidence to reject the null hypothesis as there were no significant differences between the two arms in pain and healing scores at each point of the study. However, this study provides valuable insights into the effects of diode laser and scalpel treatments on postoperative pain and healing in patients with OL. Both treatment modalities result in comparable pain levels at 24, 48 h, and 7 days postoperatively, with no statistically significant differences between groups, although the tongue exhibited the higher pain levels at 24 h. These results differed from a previous study which demonstrated inferior postoperative discomfort for patients treated with diode laser compered to scalpel after 72 h and 7 days (16). The patients of our study experienced a decrease in mean pain over time in both treatment modalities. Previous study had reported a mean pain score at first 24 h of 4 post diode laser, which is similar with our results (17). Another study with homogeneous OL treated with a diode laser showed that pain intensity was also mild and absolutely zero on the VAS scale after 1 month follow up (18). Although there was no statistical difference between two treatments, in other previously published study laser therapy has emerged as a promising alternative, offering advantages such as superior hemostasis and improved management of lesions with locations hard-to-access (19).

Pain levels were significantly influenced by the location of the lesions, with the tongue being the site associated with the highest pain scores at 24 and 48 h. This result is similar with previous literature (20). The tongue represents the most common location of OL, and the pain associated with tongue can be attributed to their constant mobility during speech and eating, as well as its contact with the dental arch and prostheses, which may exacerbate discomfort. In contrast, lesions on the palate and gingiva were associated with lower pain scores. These findings emphasize the importance of considering lesion location when planning treatment and managing postoperative care. However, it is crucial to recognize that other factors, such as lesion size, analgesic use, and adherence to postoperative instructions, may also play a role in influencing pain levels.

Our study revealed a significant negative correlation between pain levels and age, with older patients reporting lower pain scores at 24 and 48 h postoperatively. This finding aligns with previous studies, which suggest that older individuals generally perceive and report pain differently, potentially due to age-related changes in pain thresholds (21). Notably, Tighe et al. (22) highlighted that while younger patients experience higher initial pain levels, their pain resolution is more rapid within the first 24 h compared to older patients.

Healing outcomes in both treatment groups improved significantly over time, with noticeable progress from the first week to the third month. While scalpel-treated patients exhibited slightly better healing scores during the first week and first month, laser-treated patients demonstrated superior healing outcomes by the end of the three-month follow-up. This initial difference may stem from the distinct wound healing mechanisms of the two methods—scalpel surgery involves primary closure with sutures, whereas laser wounds heal by secondary intention. The laser's precise cutting and coagulative properties may minimize tissue damage, ultimately promoting smoother and more aesthetic healing in the long term (23).

Consistent with prior research, our findings underscore the advantages of laser therapy in promoting faster recovery and reducing scarring compared to scalpel methods. Studies such as those by Natekar et al. (19) and Petrov et al. (24) have demonstrated that laser treatments are associated with less postoperative pain and quicker healing. These benefits are attributed to the laser's ability to coagulate blood vessels, minimize nerve exposure, and reduce collateral tissue damage (24). While scalpel surgery may offer initial healing benefits due to sutures, the controlled nature of laser therapy appears to provide a superior long-term outcome for patients with OL.

In addition to diode lasers, other types of lasers, such as carbon dioxide (CO₂) and erbium-doped yttrium aluminum garnet (Er:YAG) lasers, have been utilized for the treatment of OL. CO₂ lasers are especially effective in managing OL due to their precision and strong hemostatic properties, which significantly reduce intraoperative bleeding and minimize scarring compared to traditional scalpel excision (15). However, as observed in our study, postoperative pain levels remain comparable between CO₂ laser and scalpel treatments (15). On the other hand, Er:YAG lasers offer the advantage of causing minimal thermal damage to surrounding tissues and have been associated with significantly lower postoperative pain scores compared to scalpel in OL treatment (25). These findings highlight the distinct benefits of laser treatments over conventional scalpel techniques, with CO₂ lasers excelling in reducing bleeding and scarring, and Er:YAG lasers providing enhanced patient comfort through reduced postoperative pain. Given these advantages, the use of lasers for the surgical treatment of OL is highly beneficial and should be considered a valuable alternative in the management of this condition.

This study has several limitations that should be considered when interpreting the results. First, the sample size may limit the generalizability of the findings to broader populations. Second, our randomization did not balance out leukoplakia locations in the two arms. Third, the assessment of postoperative pain relied on self-reported VAS scores, which are subjective and may vary based on individual pain thresholds and reporting tendencies. Fourth, we did not analyze other variables that could influence pain and healing outcomes, such as lesion size and analgesic or antibiotic use. Fifth, a potential limitation of our study is the use of the VSS to assess wound healing, as this tool was originally designed for skin rather than oral mucosal tissues. While the VSS has been applied in various contexts, including intraoral wound healing, its applicability to mucosal lesions remains suboptimal. An alternative assessment tool, the Mucosal Scarring Index (MSI), has been specifically developed to evaluate scarring in mucosal tissues and may provide a more accurate measure of post-treatment healing in oral leukoplakia (26). Future studies should consider incorporating the MSI to enhance the precision of mucosal wound assessment. Sixth, this study did not assess outcomes related to recurrence and malignant transformation. However, some participants experienced recurrence and malignant transformation, and these results have been published in a previous study (11).

In summary, our findings contribute to the growing body of evidence supporting laser excision as an effective alternative to scalpel excision for managing OL. Although no significant differences in pain levels were observed between the laser and scalpel arms in this study, the long-term healing benefits associated with laser treatment suggest its utility in improving patient outcomes. Additionally, the negative correlation between pain and older age, as well as the influence of lesion location on postoperative discomfort, highlights the need for individualized treatment approaches. Further research should explore these factors in greater depth to optimize treatment strategies for OL patients.

## Conclusion

Our study demonstrated that diode laser and scalpel treatments result in similar postoperative pain levels at 24 h, 48 h, and 7 days, with no statistically significant differences between the groups. The tongue was the location associated with the highest level of pain at 24 h. Healing outcomes were comparable, with no significant variation in contour, distortion, or texture across the evaluated time points. These findings support the use of both modalities as viable therapeutic options for OL management.

## Data Availability

The raw data supporting the conclusions of this article will be made available by the authors, without undue reservation.
